# Aqueous Interleukin-6 Levels Are Superior to Vascular Endothelial Growth Factor in Predicting Therapeutic Response to Bevacizumab in Age-Related Macular Degeneration

**DOI:** 10.1155/2014/502174

**Published:** 2014-07-10

**Authors:** Kakarla V. Chalam, Sandeep Grover, Kumar Sambhav, Sankarathi Balaiya, Ravi K. Murthy

**Affiliations:** Department of Ophthalmology, University of Florida College of Medicine, 580 West 8th Street, Tower 2, Jacksonville, FL 32209, USA

## Abstract

*Objective*. To prospectively evaluate the effect of intravitreal bevacizumab on aqueous levels of interleukin-6 (IL-6) and vascular endothelial growth factor (VEGF) in patients with exudative age-related macular degeneration (AMD) and correlate clinical outcomes with cytokine levels. *Methods*. 30 eyes of 30 patients with exudative AMD underwent intravitreal injection of bevacizumab three times at monthly intervals. The aqueous samples prior to the 1st injection (baseline) and 3rd injection were analyzed for VEGF and IL-6 levels. Subjects were subgrouped based upon change in the central subfield (CSF) macular thickness on SD-OCT at 8 weeks. Group 1 included patients (*n* = 14) with a decrease in CSF thickness greater than 10% from the baseline (improved group). Group 2 included patients (*n* = 16) who had a decrease in CSF thickness 10% or less (treatment-resistant). *Results*. In subgroup analysis, in both groups 1 and 2 patients, compared to aqueous VEGF, aqueous IL-6 levels showed a better correlation with CSF thickness on SD-OCT (*r* = 0.72 and 0.71, resp.). *Conclusions*. Aqueous IL-6 may be an important marker of treatment response or resistance in wet macular degeneration. Future therapeutic strategies may include targeted treatment against both VEGF and IL-6, in patients who do not respond to anti-VEGF treatment alone.

## 1. Introduction

Age-related macular degeneration (AMD) is the leading cause of irreversible blindness among patients over the age of 55 years in the western world [1, 2]. Exudative AMD, a less common form, is characterized by the formation of a choroidal neovascular membrane that emanates from the choriocapillaris through defective Bruch's membrane [3]. Vascular endothelial growth factor (VEGF), a diffusible cytokine that induces endothelial cell proliferation and leakage, has been implicated as an important factor in the neovascular process and associated exudation, hemorrhage, and subsequent development of detachment of the neurosensory retina and retinal pigment epithelium (RPE) [4, 5].

Inhibition of VEGF has become a widely accepted treatment modality of exudative AMD [6, 7]. Bevacizumab, an anti-VEGF agent, binds and inhibits VEGF related cellular effects [8]. In addition to VEGF, choroidal neovascularization (CNV) involves a number of angiogenic molecules and inflammatory cytokines: interleukin-6 (IL-6), interleukin-8 (IL-8), intercellular adhesion molecule-1 (ICAM-1), and monocyte chemoattractant protein-1 (MCP-1) [9, 10].

IL-6, a multifunctional cytokine, is implicated in angiogenesis along with VEGF in a variety of in vitro and cancer studies [11, 12]. High levels of IL-6 in blood have been shown to be associated with the progression of CNV [13]. Previously, we have identified the reduction in IL-6 levels (more significantly than any other cytokine) in human aqueous samples after intravitreal injection of bevacizumab in CNV associated with exudative AMD [14]. However, concurrent correlation between aqueous levels of VEGF and IL-6 and its relation to clinical outcome after intravitreal bevacizumab are not known in patients with active CNV associated with exudative AMD.

In this study, we measured IL-6 and VEGF levels in aqueous samples of patients with CNV prior to and after administration of intravitreal bevacizumab to evaluate its efficacy and attempted to evaluate relationship between the cytokine levels and clinical outcome.

## 2. Methods

In this prospective, nonrandomized, interventional study, we measured aqueous cytokines including VEGF and IL-6 in patients undergoing intravitreal anti-VEGF treatment for exudative AMD. Approval for this study was obtained from Institutional Review Board and an informed consent was obtained from all the participating subjects. The control group included 30 eyes from 30 patients undergoing cataract surgery without any other concurrent eye disease and without any prior intraocular surgery. The study group consisted of 30 eyes from 30 patients diagnosed with exudative AMD clinically and confirmed with fluorescein angiogram and spectral-domain optical coherence tomography (SD-OCT, Spectralis, Heidelberg Engineering, Heidelberg, Germany). The inclusion criteria for the study group consisted of active choroidal neovascular membrane with a washout period of 3 months from any previous treatment, age greater than 60 years, and ability to provide an informed consent. The exclusion criteria for both groups were presence of other concurrent retinal pathologies and presence of disciform scars.

All the study subjects underwent intravitreal injection of bevacizumab (Avastin: Genentech, San Francisco, CA), 1.25 mg in 0.05 mL, repeated three times at four-week interval (baseline, 4 and 8 weeks). An ophthalmic examination including recording of best-corrected Snellen visual acuity and measurement of intraocular pressure was obtained prior to first injection (baseline) and after the third injection (8 weeks). All the study patients underwent macular imaging with Spectralis OCT at the above mentioned time points. Central subfield macular thickness (CSF thickness) was obtained from the data analyzed by the machine.

## 3. Samples

For the study group, aqueous humor was obtained from 30 patients undergoing bevacizumab injections for exudative AMD. The aqueous humor samples were acquired immediately preceding the intravitreal injection. The first sample was acquired before the first intravitreal bevacizumab injection, representing the baseline sample. The second sample was obtained prior to the third intravitreal injection of bevacizumab, to evaluate the effect of the treatment with 2 doses of bevacizumab on IL-6 and VEGF levels. Aqueous humor (80–100 *μ*L) was obtained through a limbal paracentesis site using a 27-gauge needle on a tuberculin syringe. Aqueous samples were collected without touching intraocular tissues. The samples were immediately frozen to prevent protein denaturation and stored at −80°C. 30 control samples were similarly acquired from age-matched patients without any known retinal pathology, undergoing cataract surgery.

## 4. Multiplex Analysis

Multiplex analysis was preformed to discern biomarker patterns within small sample volumes. The aqueous samples were thawed at room temperature, vortexed, and then spun at 1400 rpm for 10 min to remove any precipitates. Subsequently, samples were diluted 1 : 3 and processed for IL-6 and VEGF analysis using singleplex bead immunoassay (Invitrogen Corporation, US). Standards were reconstituted and serially diluted, per the manufacturer's instructions. A 96-well microtiter plate was used and 25 *μ*L of bead solution was added to each designated well and incubated for 30 seconds. After incubation, the supernatant was aspirated, using a vacuum manifold; 50 *μ*L of incubation buffer was added to each well prior to 50 *μ*L of assay diluent and 50 *μ*L of the aqueous sample. Diluted standards were then added to the designated wells and the plate was then incubated for 2 hours at room temperature on an orbital shaker (250–500 rpm). After incubation, the liquid was removed and the excess beads were washed repeatedly. Furthermore, 100 *μ*L of detector antibody, specific for VEGF and IL-6, was added to each well. The plate was incubated on an orbital shaker at room temperature for an hour. After incubation, excess detector antibodies were removed and substrate complex (S-RPE: Streptavidin-R-Phycoerythrin) was added. Luminex 100 IS fluoroanalyzer (Luminex Inc., Austin, TX, US) was used to measure the fluorescence after 30 minutes of incubation at room temperature. To ensure proper assay, a known concentration of human recombinant VEGF as well as IL-6 was included in each run as a positive control.

The time points in the study were at baseline and at 8 weeks. The primary outcomes of this experiment were measurement of aqueous VEGF and IL-6 concentrations in the study group before and after treatment with 2 doses of intravitreal bevacizumab. The secondary outcomes consisted of correlation of change in aqueous VEGF and IL-6 concentrations with change in visual acuity and CSF thickness.

## 5. Statistics

Statistical analysis was performed with GraphPad Instat (GraphPad Software, Inc., CA, US). Snellen visual acuity was converted to logMar for analysis. Baseline parameters were compared using the Chi-square test; changes in aqueous VEGF and IL-6 values (at baseline and at 8 weeks) were analyzed using Wilcoxon rank sum test and correlation statistics were performed using Spearman correlation test.

## 6. Results

Baseline characteristics of the control and study patients are as shown in Table 1. Thirty patients were included in the control group with a mean age of 72.8 ± 7.0 years. 30 patients diagnosed with exudative AMD formed the study group with a mean age of 73.7 ± 8.6 years. The study group had predominantly male patients (female/male 7 : 23) and patients were predominantly Caucasian (African American/Caucasian 1 : 29).

Study patients had significantly higher VEGF and IL-6 levels compared to controls. Mean aqueous VEGF concentration in the study group was 6 ± 3 picograms (pg) compared to 3.7 ± 1.3 pg in controls (*P* = 0.001). Similarly, mean aqueous IL-6 concentration in the study group was 17.1 ± 21.3 pg compared to 5.1 ± 2.2 pg in controls (*P* < 0.0001).

In the study, best-corrected visual acuity at baseline in the study population was 0.52 ± 0.65 logMar and at 8 weeks it was 0.58 ± 0.62 logMar (*P* = 0.43). Mean baseline intraocular pressure was 14.3 ± 3.4 mm Hg and following treatment was 13.9 ± 3.7 mm Hg (*P* = 0.5).

The mean baseline CSF thickness was 443.7 ± 215.7 microns and at 8 weeks it was 370.5 ± 143.6 microns representing a change of 11.5 ± 20.8% (*P* = 0.009). Visual acuity correlated poorly with change in CSF thickness (*r* = 0.01).

**Figure 1:**
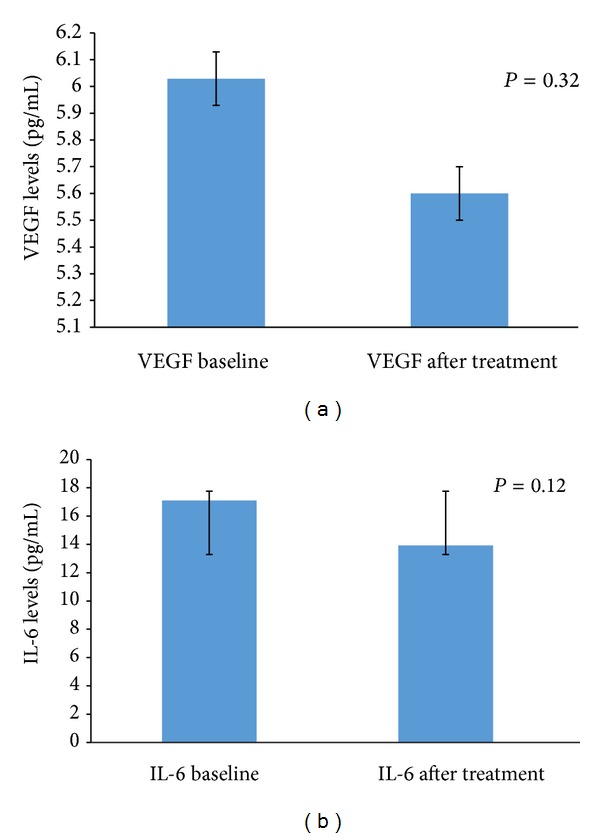
Column chart showing the change in mean aqueous VEGF levels (a) and change in mean aqueous IL-6 levels (b) in the study patients. The error bar represents standard deviation.

There was no significant difference in aqueous VEGF levels (*P* = 0.38) between baseline and 8 weeks after injection, although levels decreased in most cases and a decreasing trend was discernible (Figure 1(a)). Posttreatment aqueous VEGF levels showed poor correlation with change in CSF thickness (*r* = 0.19, *P* = 0.32). Similar to aqueous VEGF concentration, there was no significant difference between baseline and 8 weeks after injection in aqueous IL-6 levels (*P* = 0.12) (Figure 1(b)). Similar to aqueous VEGF, there was a decreasing trend in aqueous IL-6 levels observed following treatment. However, change in aqueous IL-6 levels showed significant correlation with change in CSF thickness (*r* = 0.64; *P* = 0.002).

Analyses of data revealed that there was no uniform resolution of macular thickening on Spectralis OCT following intravitreal bevacizumab treatment. The study patients were then subgrouped based on their CSF thickness change. Group 1 included patients (*n* = 14) who had a decrease in CSF thickness greater than 10% from the baseline and were categorized to have “improved.” Group 2 included patients (*n* = 16) who had a decrease in CSF thickness 10% or less and were considered “treatment-resistant.”

**Figure 2:**
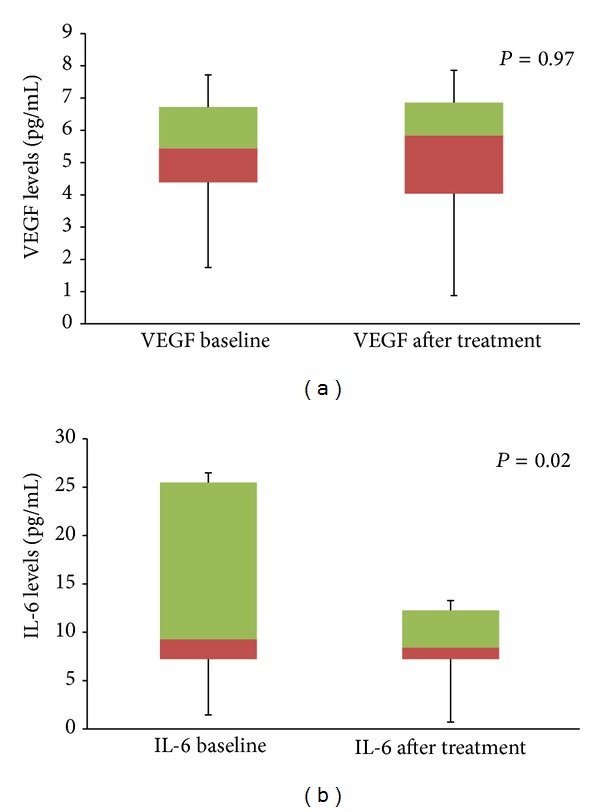
Box plot diagram showing the change in mean aqueous VEGF levels (a) and change in mean aqueous IL-6 levels (b) in patients in group 1. The error bar represents standard deviation.

In the subanalysis, in group 1 patients, mean change in visual acuity was 0.0 ± 0.27 logMar. Mean CSF thickness at baseline was 506.7± 238.5 microns and at 8 weeks was 362.6 ± 133.1 microns (*P* = 0.001). Aqueous VEGF concentrations at 8 weeks were 5.6 ± 2.1 pg compared to 5.7 ± 2.1 pg at baseline (*P* = 0.97) (Figure 2(a)). Similarly, aqueous IL-6 concentration at 8 weeks was 12.7 ± 7.2 pg compared to 23.3 ± 25.9 pg at baseline (*P* = 0.02) (Figure 2(b)). There was a stronger correlation of change in CSF thickness with aqueous IL-6 levels (*r* = 0.72) compared with aqueous VEGF levels (*r* = 0.42) (Figure 4).

**Figure 3:**
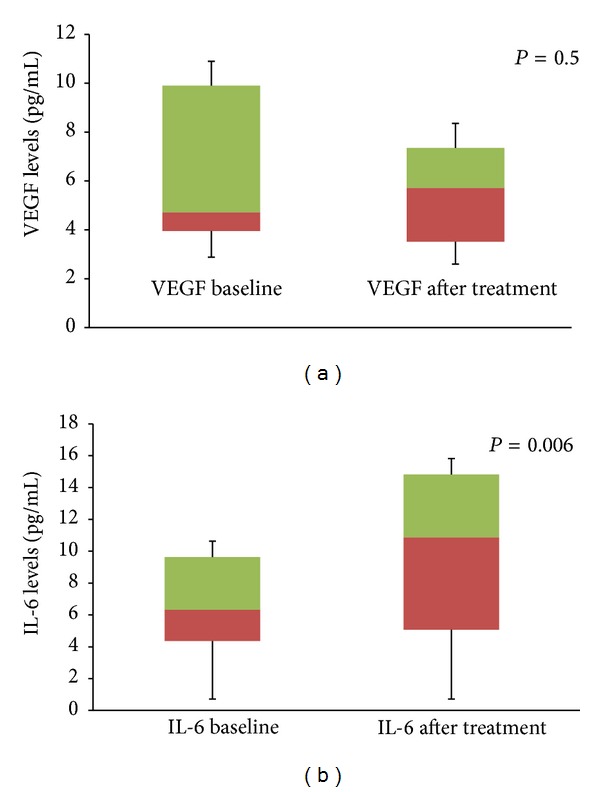
Box plot diagram showing the change in mean aqueous VEGF levels (a) and change in mean aqueous IL-6 levels (b) in patients in group 2. The error bar represents standard deviation.

**Figure 4:**
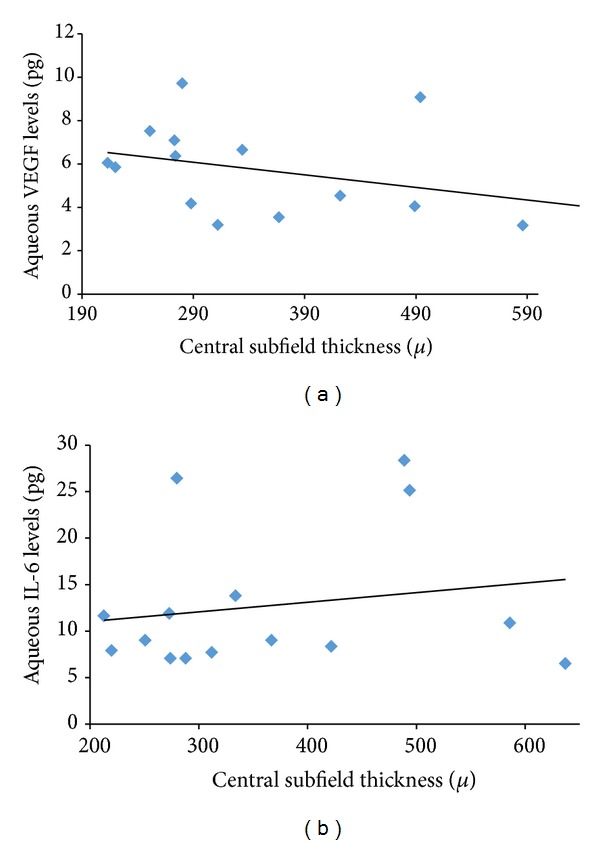
Correlation plot showing correlation between aqueous VEGF (a) and aqueous IL-6 levels (b) with central subfield thickness on Spectralis OCT in patients in group 1.

**Figure 5:**
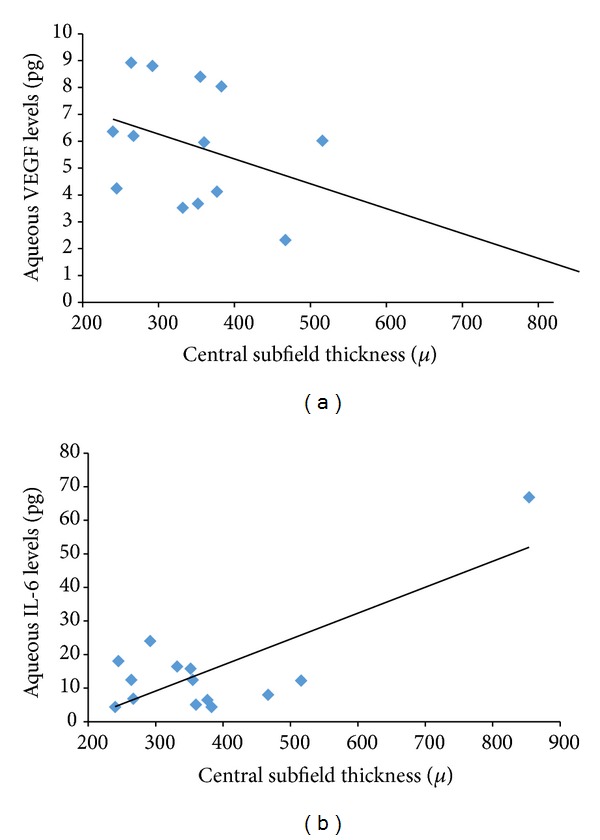
Correlation plot showing correlation between aqueous VEGF (a) and aqueous IL-6 levels (b) with central subfield thickness on Spectralis OCT in patients in group 2.

In group 2 patients, mean change in visual acuity was −0.14 ± 0.61 logMar. Mean CSF thickness before treatment was 376.2 ± 171.6 microns and at 8 weeks was 378.9 ± 158.5 microns (*P* = 0.1). Aqueous VEGF concentrations were 5.5 ± 2.5 pg compared to 6.5 ± 3.7 pg at baseline (*P* = 0.5) (Figure 3(a)). Similarly, aqueous IL-6 concentration was 14.6 ± 15.5 pg compared to 10.4 ± 12.8 pg at baseline (*P* = 0.006) (Figure 3(b)). Similar to group 1, IL-6 showed stronger correlation with change in CSF thickness compared to aqueous VEGF levels (*r* = 0.53 and 0.79, resp.) (Figure 5).

## 7. Discussion

In this study, we investigated the correlation between VEGF and IL-6 levels in the aqueous with the treatment response after intravitreal bevacizumab for exudative AMD. Aqueous IL-6 levels showed a better correlation than aqueous VEGF levels in predicting treatment response in exudative AMD after bevacizumab treatment.

Exudative AMD characterized by choroidal neovascularization is driven by ischemia induced upregulation of VEGF [4]. Increased levels of VEGF in the aqueous are observed in several ocular ischemic conditions including diabetic retinopathy, retinal vascular occlusions, and exudative AMD [5]. In our study, we measured cytokines level using bead-based immunoassay with flow cytometry, which we previously have shown to accurately measure VEGF concentration in microsamples [15]. Besides ischemia, inflammation has been implicated in the etiopathogenesis of CNV [9]. IL-6, a key inflammatory mediator, is a multifunctional cytokine that can indirectly increase vascular permeability by inducing VEGF expression and directly increase endothelial permeability [11]. Similar to VEGF, aqueous IL-6 levels have been found to be increased in patients with ischemic retinal conditions [16]. In patients with active CNV, Roh et al. have reported significant correlation between the aqueous humor levels of IL-6 and the size of CNV [17]. However, in their study, IL-6 levels in the aqueous measured in patients with treatment-naïve CNV, patients with recurrent CNV, and patients with regressed CNV were not significantly different from that measured in the control group.

Aqueous VEGF levels have been shown to normalize after intravitreal bevacizumab in retinal conditions such as diabetic macular edema and retinal vascular occlusions [18]. However, similar correlation has not been established in exudative AMD. In our study, no statistical significant reduction in aqueous VEGF and IL-6 levels was found after intravitreal bevacizumab treatment, though both cytokine levels showed a decreasing trend. Interestingly, in the treatment response group there was normalization of IL-6 levels (levels comparable to the IL-6 levels in the control group) indicating that IL-6 is a better marker of disease activity. Roh et al. have reported that the concentration of aqueous cytokines (including IL-2, 4, 6, 8, 10, TNF-*α*) was found to be unchanged after consecutive intravitreal bevacizumab [19]. Focality of disease process in AMD is one possible explanation for the poor correlation of aqueous VEGF levels with the treatment response.

In ischemic retinal conditions, aqueous VEGF levels have been shown to correlate with the severity of the disease process, which is measured clinically with visual acuity or the macular thickness on OCT [16]. In the study, treatment response was defined based on the change in central macular thickness noted on sequential scans done at 8 weeks apart on Spectralis OCT. Studies correlating visual acuity outcomes after anti-VEGF treatment with retinal parameters on SD-OCT have shown that central retinal thickness correlates poorly with visual acuity [20]. Retinal scans obtained on SD-OCT have been further delineated using segmentation algorithms to show that visual acuity correlates better with outer retinal layer thickness in the fovea after anti-VEGF treatment in patients with exudative AMD [21]. However, these segmentation algorithms are not uniformly applied in clinical practice. In our study, we used central subfield macular thickness (CSF thickness), which is the average thickness measured within the inner circle of 1 mm diameter, to follow response to treatment. Previously, studies reporting normative data on macular thickness have reported high repeatability of CSF thickness with a coefficient correlation less than 9%, with values above it representing a true onset of macular thickening [22]. Extrapolating from the same guideline for exudative AMD, we categorized treatment response to intravitreal bevacizumab as a decrease in CSF thickness of more than 10% from baseline and a treatment-resistant group as a decrease in CSF thickness of 10% or less. In our study, a statistically significant correlation was found between IL-6 levels and CSF thickness in both treatment-responsive and treatment-resistant groups, with stronger correlation in patients who showed treatment-resistance with intravitreal bevacizumab. On the other hand, no significant correlation was found in aqueous VEGF and CSF thickness in either group. The exact mechanism for increase in aqueous IL-6 levels after intravitreal bevacizumab cannot be explained. However, we hypothesize that there was upregulation of the pathway upstream (principally the inflammatory cytokines such as IL-6) as bevacizumab neutralized the VEGF present intraocularly.

The strengths of our study include larger patient population, uniformity of treatment protocol, and use of sensitive assays for measuring cytokines.

In conclusion, data from our study suggests that aqueous IL-6 may be an important marker of treatment response. Future therapeutic strategies may include targeted treatment against both VEGF and IL-6 in patients who do not respond to anti-VEGF treatment alone.

## Figures and Tables

**Table 1:** Baseline characteristics of study and control population.

	Control group	Study group	*P* value
Age (in years)	72.8 ± 7.0	73.7 ± 8.6	0.2

Sex (female : male)	4 : 26	7 : 23	0.1

Race (African American : Caucasian)	26 : 4	1 : 29	0.001

Aqueous VEGF levels (in picogram)	3.7 ± 1.3	6 ± 3	0.002

Aqueous IL-6 levels (in picogram)	5.1 ± 2.2	17.1 ± 21.3	<0.0001
